# An Evaluation of an Online Training Platform for Teaching Positive Emotions for People With Schizophrenia

**DOI:** 10.3389/fpsyt.2022.798019

**Published:** 2022-02-07

**Authors:** Alexandra Nguyen, Laurent Frobert, Aurélien Kollbrunner, Jérôme Favrod

**Affiliations:** School of Nursing, La Source, University of Applied Sciences and Arts of Western Switzerland, Lausanne (HES-SO), Lausanne, Switzerland

**Keywords:** e-learning, anhedonia, apathy, schizophrenia, motivation, pleasure, partnership, learning strategies

## Abstract

**Background:**

The dissemination of new interventions in clinical practice remains challenging. E-learning may provide wide access in various settings and allow tailored learning trajectories and an adapted training pace. This study evaluates an online platform to train professionals to lead the Positive Emotion Program for Schizophrenia (PEPS) for patients with anhedonia. This study aims to test the reception provided by clinicians to the platform and its perceived usefulness and investigate whether e-PEPS training improves knowledge about the facilitation of PEPS.

**Materials and Methods:**

Participants were recruited through advertisements. All participants provided their informed consent on a registration form and completed two pre-test questionnaires, a knowledge test on negative symptoms in schizophrenia, learning strategies and the partnership relationship, and a test on the ability to savor pleasant moments. After the training, they completed the same questionnaire and an evaluation form of the training and its application in personal and professional life.

**Results:**

Two-hundred and ten participants were registered to participate into the study, 185 received the access to the platform, and 101 participants completed the training and the post-test assessments. Satisfaction with training was high. The results showed that the participants significantly improved their knowledge about PEPS and increased the skills taught in their personal repertoire after the training. The training allows most clinicians to plan to lead a PEPS group in the year following training.

**Discussion:**

As a result of this study, training has been improved and is now freely available to all interested clinicians.

## Introduction

Anhedonia and avolition hamper the quality of life and functioning in people with schizophrenia, and the efficacy of drug-based treatments and psychological interventions on primary negative symptoms remain limited (1). The Positive Emotions Program for Schizophrenia (PEPS) has been developed to target these symptoms by increasing the anticipation and maintenance of positive emotions and beliefs about future performance (2). PEPS involves eight 1-h group sessions administered using visual and loud audio materials and presented as PowerPoint presentation slides projected onto a screen. The program uses a collaborative egalitarian approach. A pilot study indicated that PEPS is both a feasible intervention and is associated with a specific reduction in anhedonia and apathy (3). A randomized controlled trial, in which raters were blinded to the conditions under which participants were randomized, was conducted and analyzed using an intention-to-treat analysis. Results showed statistically significant clinical improvement in PEPS participants compared with non-PEPS participants at post-test and 6-month follow-up assessments for the apathy and anhedonia composite scores on the Negative Symptom Rating Scale (4). Finally, a field test showed that PEPS could be easily administered after a day of training, with a reduction in negative symptoms and an improvement in social functioning in patients (5).

The dissemination of new interventions in clinical practice remains challenging. Traditionally, new psychosocial interventions are presented face-to-face and supplemented with manuals and clinical supervision. This training method places a high demand on human resources because the developers of new interventions may be involved in clinical practice and may want to pursue their research and development work. The number of participants will be limited because of geographical factors and limited class sizes to teach practical skills (6). E-learning may help overcome these obstacles, allowing extensive access in various settings (home, work, mobile phones), tailored learning trajectories and adapted training pace. The lack of peer support may be overcome by suggesting online teams. Creating an online community of practice may also allow us to surmount feelings of isolation or lack of peer support.

e-PEPS, an online training platform that prepares healthcare and social work professionals to run the Positive Emotions Program for Schizophrenia (PEPS), was created to address the aforementioned challenges. e-PEPS is intended to help these professionals achieve the following objectives:

- Become familiar with the theoretical and clinical foundations of PEPS to promote the development of positive emotions in participants.
- Identify learning strategies and mobilize educational strategies to support the learning of participants in an individualized manner.
- Strengthen the partnership posture between facilitators and participants to ensure regular participation in sessions, reduce stigma and self-stigma, and strengthen the therapeutic alliance.

The program consists of three modules that address these three objectives. On the home page, there is advice on building one's own training pathway based on prior knowledge or learning style. The main content is brought through video clips, which include theoretical videos presenting the concepts underlying the design of PEPS clinically and pedagogically, demonstration videos featuring animation sequences of the program, training videos to identify significant elements for the conduction of the program, and reflective videos to analyze practices by highlighting significant elements in the facilitation of sessions. Quizzes, reflective activities, and practice exercises are offered or integrated into videos, making them interactive. The downloadable texts and articles completed the training.

This evaluation of e-PEPS online training on professional aspects and competences aimed to test the reception provided by clinicians to the platform and its perceived usefulness. It will investigate whether e-PEPS improves knowledge score about the animation of PEPS and their dispositional beliefs about their ability to appreciate positive experience. The study will also measure how the clinician use the skills taught in their repertoire and apply them in their actual clinical practice.

## Materials and Methods

The e-PEPS online training was evaluated between September 15 and December 15, 2020. Participants were recruited through the Swiss Society of Social Psychiatry, the International Association of Schizophrenia and Mental Health Days and the popular magazine “Santé mentale,” a monthly magazine for psychiatric care teams. They gave their informed consent on a registration form and completed two pre-test questionnaires, a knowledge test on negative symptoms in schizophrenia, learning strategies, and the partnership relationship, and a test on the ability to savor pleasant moments, the Savoring Belief Inventory (7, 8). After the training, the participants completed the same questionnaires and an evaluation form of the training and its application in personal and professional life. They received only one reminder message. Once the file was complete, the participants received a message to log into the training. A reminder message was sent 1 month later to complete the post-test questionnaires.

### Instruments

The knowledge assessment questionnaire measures knowledge about negative symptoms, skills taught in PEPS, learning strategies, partnership relationships, and support strategies used in program facilitation. It consists of 14 multiple-choice questions. A score between two and six points was given for each question for a total of 41 points (7 2-point questions, 3 3-point questions, 3 4-point questions and 1 6-point question). The knowledge questionnaire was tested by comparing 11 people trained to facilitate PEPS with 12 people who were unfamiliar with the program. Experienced people obtained an average score of 29.91 (SD 3.9), and novices had an average score of 11.67 (SD 6.53), and their score was statistically significant in this test compared to novices [*t*_(18.23)_, *p* = 0.000]. To assess the stability of the test, eleven participants took the test 15 days apart; the test was particularly stable [Spearman's rho = 0.75, *p* = 0.008; *t*_(10)_ = 0.000, *p* = 1.00].

The French version of the Savoring Beliefs Inventory (SBI) (8) is a self-assessment questionnaire composed of 24 items divided into three-time dimensions: past, present, and future each represented by eight items. It is a self-report measure of people's dispositional beliefs about their ability to appreciate positive experience in each of these three temporal (9). Half of the items are formulated positively, while the other half are formulated negatively. Each item is scored on a 7-point Likert scale, ranging from “strongly disagree” to “strongly agree”. The total score of the SBI is calculated by subtracting the total score of items framed negatively from the total score of items formulated positively. The three subscales: the anticipation of pleasure, pleasure in the present moment, and recalled pleasure from a positive experience, are calculated similarly. The anticipation of pleasure subscale measures the ability to savor a positive future event in advance, the present moment pleasure subscale measures the enjoyment of positive events when they occur, and the remembered pleasure subscale measures the recall of positive past events after they have occurred. The original English version of the SBI was independently translated by three native French speakers and compared until full agreement was reached. The translation was authorized by the author of the original version. The factor structure of the French version of the SBI was adequate, and all items contributed significantly to their corresponding factor: Anticipating pleasure, Present moment pleasure, and Reminiscing pleasure (8).

The post-test evaluation questionnaire is composed of 10 items that evaluate the interest, usefulness, achievement of objectives, navigability, coherence of training in a global way, and specific criteria by type of video and educational activities. Twelve items evaluated learning in personal and professional life. Respondents could make comments and suggestions. Each item is evaluated on a 4-points Likert scale (Fully disagree, partially disagree, partially agree, fully agree).

### The Intervention e-PEPS

The training is available at https://www.e-peps.ch. It is recognized by the Swiss Society of Social Psychiatry for 10 h of continuing education upon successful completion of a questionnaire at the end of the training, which attests to the acquisition of knowledge to facilitate PEPS. During the present study, the project was on a development platform, and access was given under registration. The platform was built with LMS LearnDash on WordPress. The e-PEPS has a simple architecture that allows easy navigation for the participants. The five main tabs help to find the way around the platform. The home page of the site is accessible to everyone. It provides general information on the use of the site and directs the training registration link. The training tab leads to three modules that constitute the training. A PEPS tab leads to a download link to obtain the PEPS program. A PEPS community tab allows the users to register for the discussion via a forum. Finally, an account tab allows the user to configure its personal account.

**Figure 1:**
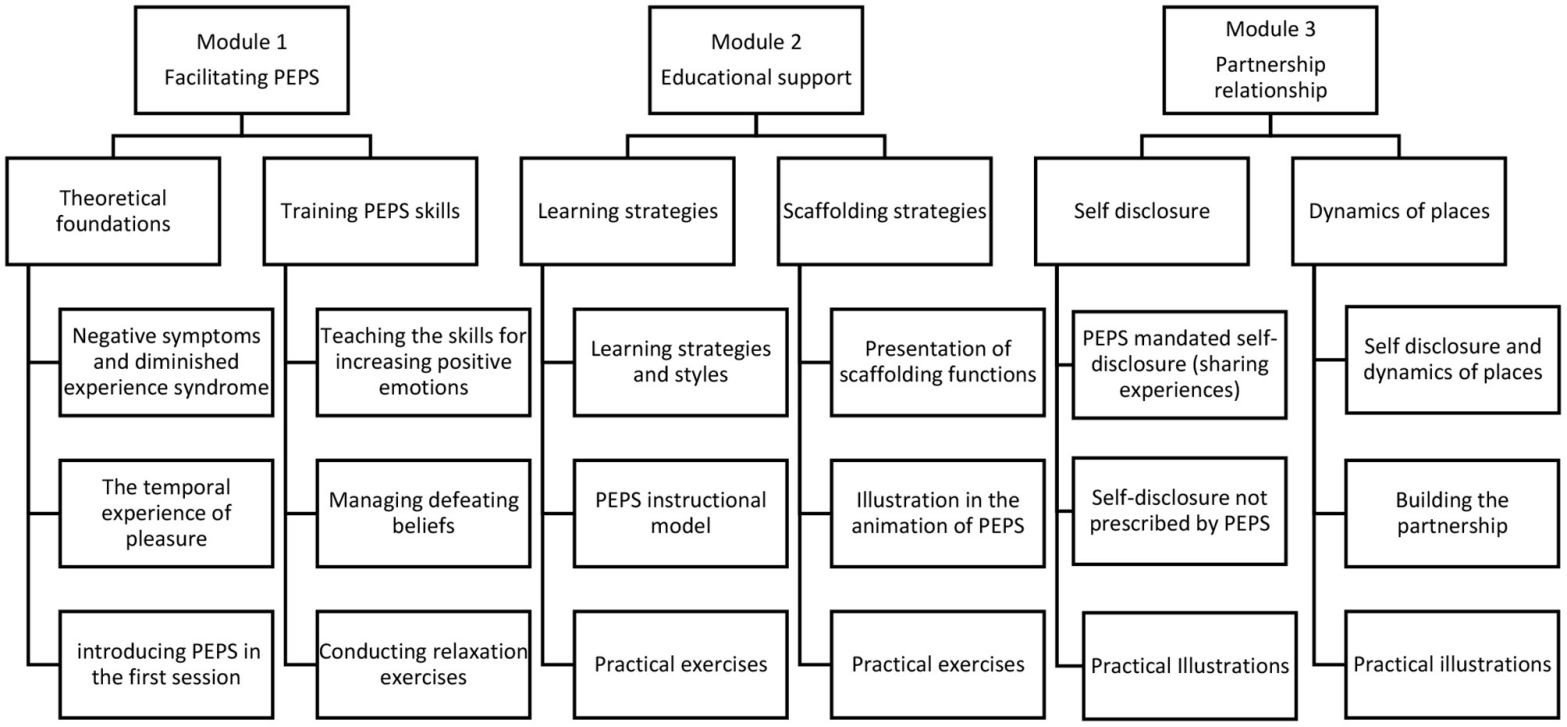
Stage by stage content of the training.

The training was organized into three modules: module 1 is training for the animation of PEPS, module 2 develops skills for pedagogical support, and module 3 teaches the principles of the partnership relationship (see Figure 1). The three modules of training are divided into sequences and activities. Module 1 presents the theoretical foundations of the program and demonstration videos in a concrete manner with presentations of sequences between facilitators and participants. The theoretical contributions are supplemented by a range of scientific and professional articles. The second part of this module presents the PEPS skills: savoring pleasant experiences, accentuating the expression of emotions, capitalizing on positive moments by sharing them with others, and anticipating pleasant moments. The concepts underlying these skills are presented in a video capsule, and each PEPS skill is illustrated in the animation of a session of the program. Practical exercises are also offered to practice and test knowledge. Learners are encouraged to watch a video of a PEPS session on interactive videos. The third sequence of module 1 presents PEPS exercices that aim to reduce at reducing defeatist beliefs. As in the previous sequences, a theoretical video capsule explains the influence of defeatist beliefs on motivation and the difficulty in achieving goal-directed behaviors. Two other activities are offered to learners to work on those beliefs. A video shows the steps to help PEPS participants identify and modify these beliefs. An interactive video then suggests practicing the same skills. The fourth sequence of this module introduces relaxation exercises, explains the value of relaxation in managing emotions, and demonstrates simple exercises. Learners can practice exercising using a recorder. Module 2 focuses on educational support. The first sequence familiarizes learners with theoretical contributions resulting from experiential learning from the work of Kolb and Kolb (10). It emphasizes the four main learning strategies described in the model and helps understand how they are inserted into the PEPS program. Knowledge of learning strategies is useful for facilitators to identify those of the participants and to better support them in their learning during PEPS exercises. Again, theoretical and practical videos are used to develop the skills to accompany patients who follow PEPS according to their learning strategies and learning styles. Sequence 2 of this module presents Jerome Brunner's scaffolding strategies, again with theoretical and concrete exercises. Module 3 focuses on partnership relationships. It is divided into two sequences: one on self-disclosure practice and the other on the phenomenon of status, roles, and places in the therapeutic relationship. As the animation of PEPS requires facilitators' personal involvement, facilitators participate in sharing their experiences during exercises along with patients. They show how to describe life experiences related to PEPS skills and actively engage patients in the exercises. In sequence 2 of this third module, participants explored the dynamics of places in the therapeutic relationship. The sharing of personal experiences prescribed by the program prompts the facilitators not only to lead the group in PEPS learning activities but also to take on the role of participants, beyond the institutional functions (educator, nurse). This prescription of egalitarian participation acts on the relationships between places and favors the empowerment of the participants (11, 12). This sequence offers a theoretical description of this interactional phenomenon using a video capsule and practical illustrations of the posture valued by the program. Participants learn about the different facets of the therapeutic relationship at the deontic, epistemic, and affective levels.

### Ethical Considerations

Interested persons had to register for the training test via an online questionnaire and respond positively to the informed consent: I am interested in participating in the test of this training and give my consent so that the data collected can be used for this evaluation. The data were processed anonymously. A detailed description of the study was available on the websites of the study. This study is outside the scope of the Swiss Human Research Act because no personal data concerning human diseases and the structure and function of the human body were collected. Therefore, this study did not need to be authorized by Swiss ethics.

### Statistical Analysis

Descriptive statistics were used for statistical analysis. Pre- and post comparisons were made using a paired-sample *t*-test. Correlations were calculated using Spearman's rho rank correlation. The differences in scores were calculated by subtracting the post-test from the pre-test. Cohen's *d* effect sizes were calculated for within-subjects, correcting for dependence among means to make direct comparisons with effect sizes from between-patient studies. Formula 8 of Morris and DeShon was used (13).

## Results

**Figure 2:**
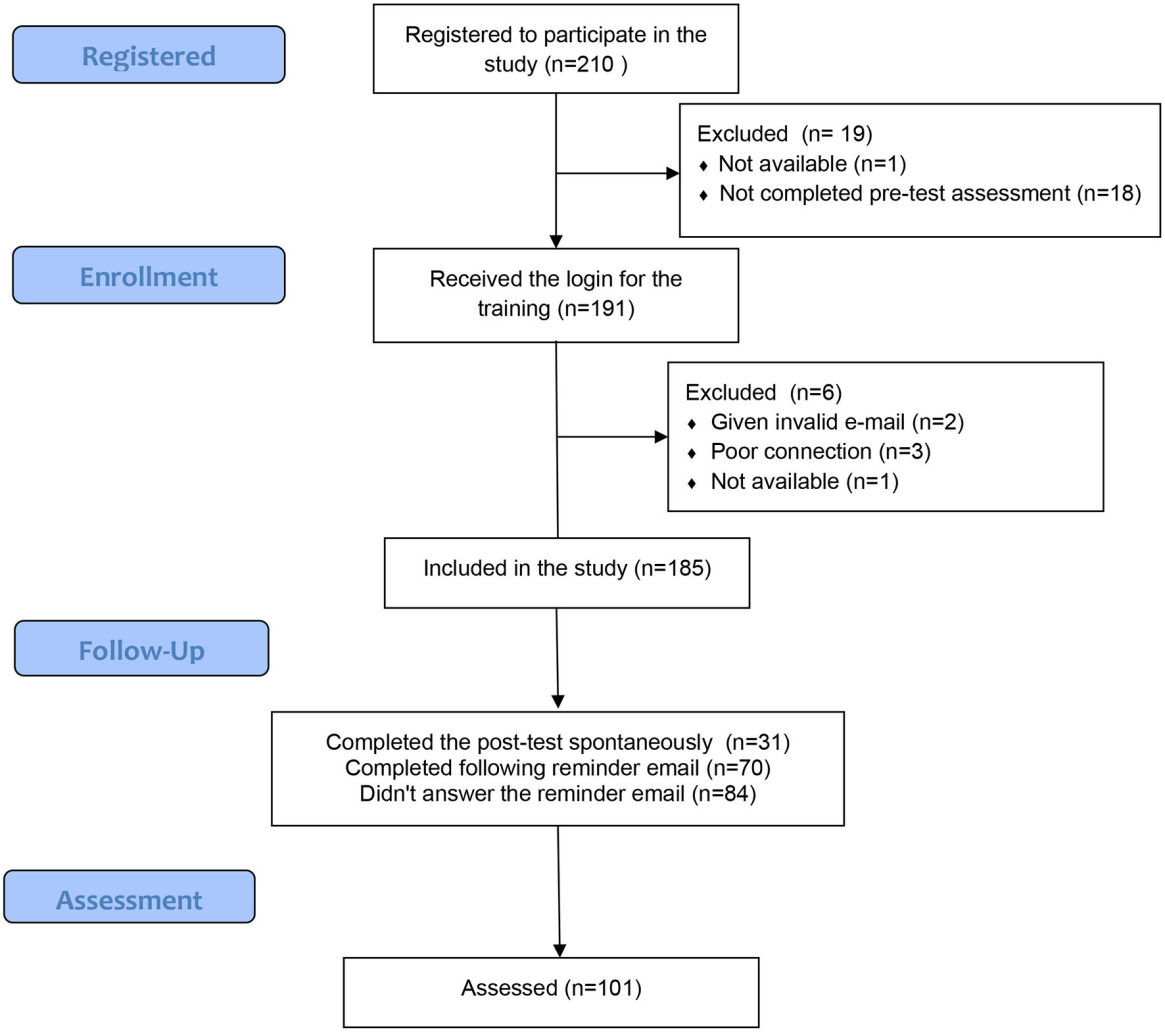
Study flow chart.

The flowchart of the study showed that 210 participants registered to participate in the study (Figure 2). One participant was no longer available a few days after registration because hired on the front line for the second wave of the COVID-19 pandemic. Eighteen participants did not complete the pre-test questionnaires and did not respond to the message reminder. One hundred and ninety-one persons had a complete file and received access to online training. Two of these 191 people had given invalid e-mail addresses and could not receive their login message. Three encountered video streaming problems related to their Internet connection. Finally, a second person was hired on the front line of the second wave of COVID-19. Thus, 185 participants could follow the training. One hundred and one participants completed the training. The sample consisted of 101 people, 88 women and 13 men, with an average age of 35.12 years (e-t. 10.02). The participants were psychologists (*n* = 28), registered nurses (*n* = 26), clinical bachelor student nurses (*n* = 17), social workers (*n* = 15), occupational therapists (*n* = 6), psychiatrists (*n* = 4), peer practitioners (*n* = 3), social science teacher (*n* = 1), and medical secretaries (*n* = 1). Twenty-five of those who completed the training worked in socio-therapeutic or nursing homes, 19 in day centers, 13 in hospitals, 12 in outpatient clinics, 8 in mobile teams, 1 in sheltered workshops, 2 were unemployed, and 3 worked in university and 17 students were in 8-weeks clinical internship. Fifty-five lived in Switzerland, 44 in France, two in Belgium, and two in Martinique.

People who took the training estimate that they completed it in an average of 8 h and 33 min (SD 3 h 42) over an average period of 29 days (SD 24 days). The results showed that the participants who took the training significantly improved their results on the knowledge test [*t*_(100)_ = −18.01; *p* = 0.000] by doubling their scores (Table 1). Thus, they join the scores of the facilitators already trained. They also statistically significantly improved their ability to savor the future [*t*_(100)_ = −3.54, *p* = 0.001], present [*t*_(100)_ = – 2.87, *p* = 0.005], past [*t*_(100)_ = −3.50, *p* = 0.001], and total SBI score [*t*_(100)_ = −4.35, *p* < 0.000] measured using the Savoring Belief Inventory-French version.

**Table 1:** Pre and post-tests results.

	**Pre-test mean (SD)**	**Post-test mean (SD)**	***T*** **(df),** ***p***	**Cohen's d**
Knowledge test about PEPS	15.47 (7.03)	29.56 (7.41)	*t*_(100)_ = −18.01; *p* = 0.000	1.80
SBI anticipation of pleasure	5.84 (0.73)	6.06 (0.68)	*t*_(100)_ = −3.54, *p* = 0.001	0.35
SBI pleasure in the present moment	5.29 (0.96)	5.47 (0.96)	*t*_(100)_ = −2.87, *p* = 0.005	0.29
SBI recalled pleasure	5.88 (0.74)	6.07 (0.67)	*t*_(100)_ = −3.50, *p* = 0.001	0.35
SBI total	17.01 (2.12)	17.60 (2.02)	*t*_(100)_ = −4.35, *p* = 0.000	0.43

*SBI, savoring beliefs inventory; SD, standard deviation*.

Check for difference between sex or correlation with age for the different variables have been explored but no differences or correlation were observed. On the other hand, we observed a slight correlation between the duration of training estimated by the participants and the improvements in the knowledge test (Spearman's rho 0.23, two-sided *p* = 0.02), on the SBI ability to savor the future (Spearman's rho 0.33, two-tailed *p* = 0.001), and SBI total score (Spearman's rho 0.26, two-tailed *p* = 0.009). Satisfaction with the training was high (see Table 2). The results of the satisfaction questionnaire and specific users' comments could be considered to improve the platform. Two videos had to be re-recorded, and some presentation texts were clarified. Four recorded situations with students could not be turned around and were subtitled to work around sound problems.

**Table 2:** Assessment of the satisfaction with the platform e-PEPS (*n* = 101).

	**Disagree**	**Partially disagree**	**Partially agree**	**Fully agree**
Overall, this e-PEPS course met my expectations			40.6%	59.4%
I find the content of the training useful		1%	19.8%	79.2%
The objectives of the training are clearly presented		4%	21.8%	74.3%
The content of the training is coherent with the announced objectives			19.8%	80.2%
The objectives of the training are achieved			47.5%	52.5%
The training is structured and well-organized		1%	31.7%	67.3%
The videos proposed facilitate learning		3%	27.7%	69.3%
Reflective activities and practical exercises contribute to learning			30.7%	69.3%
The site is easy to navigate		3%	47.5%	49.5%
I will recommend this e-learning course to other			22.8%	77.2%

As a result of the training, the participants made more use of the skills taught in PEPS in their personal lives, 82% to savor the pleasant experience, 66% to accentuate the expression of positive emotions, 70% to relate more pleasant experiences to others, 66% to anticipate good times, 48% to use the calm crisis, and 79% to manage my defeatist beliefs.

Regarding the transferability of learning in practice, 23 participants were excluded from this analysis because they were either students who completed 8-week clinical practice placements alternating with their course, unemployed, or with no clinical practice. These participants could not implement groups in the clinical practice.

Twenty of the 78 clinicians fully agreed that they felt capable of putting PEPS into practice in their field, 48 partially agreed, and 10 did partially disagree. To the question, ”I have planned to set up PEPS groups” 34 participants answered not now, 21 within a year, 8 in the next 6 months and 15 in the next 3 months. The ability to put PEPS into practice in the field is highly correlated with the feeling of competence to be able to lead PEPS (Spearman's rho 0.71, *p* = 0.000) as well as the pedagogical skills acquired (Spearman's rho 0.50, *p* = 0.000), skills acquired in participating as a model for patients (Spearman's rho 0.27, *p* = 0.02), and the ability to share their own experiences in the therapeutic relationships (Spearman rho 0.33, *p* = 003).

Fifty-five participants in this sample of clinicians believe that their work context facilitates the establishment of a PEPS group. It appears that 84% of the participants working in a day program, 75% in outpatient clinics, 72%, in socio-therapeutic or nursing homes and 54% in hospitals and 50% in mobile teams see their clinical field as facilitating to lead a PEPS group.

## Discussion

This evaluation of e-PEPS online training on professional aspects and competencies aimed to test the reception given to the platform by clinicians and its perceived usefulness. Of the 185 available participants, 56% completed the training. In comparison, a review of completion rates for 221 Massive Open Online Courses show variation from 0.7 to 52.1%, with a median value of 12.6% (14). A major challenge of e-learning courses is to keep students motivated to complete the training (15, 16). Satisfaction with the training is high with a wide and varied audience from various health and social professions. It is also a pre and post-test study to investigate whether e-PEPS improves knowledge about the facilitation of PEPS, the use of skills taught in the clinicians' own repertoire, and application in actual clinical practice. The results showed that the participants significantly improved their knowledge about PEPS and increased the use of the skills taught in their personal repertoire after the training. The training allows most clinicians to plan to lead a PEPS group in the year following the training. The best places to run PEPS groups seem to be clinical settings where contact with patients is easier to plan, such as day programs or outpatient clinics, as well as socio-therapeutic or nursing homes. Implementation of psychosocial interventions in psychiatry remains a major challenge and translating knowledge into practice can take years and even decades (17). Availability of training and materials is a first condition. Training tends to improve attendees' knowledge, attitudes, and confidence in working with clients (18) but a training-only approach has not demonstrated effectiveness in changing provider behavior (19). Only a small proportion of our sample feel capable to running PEPS in clinical practice and this seems to be consistent with the data in the field. The main advantage of e-PEPS over live training is that participants can return to the online training to learn and update their knowledge at any time. Follow-up consultation, supervision, or feedback are recommended for long-term adoption of skills, but even such attempts to promote adoption of evidence base psychosocial intervention in community mental health clinics can be deceiving (19). To support the adoption and maintenance of new therapeutic approaches, it is also important to overcome organizational barriers. Organizations' willingness to bring the best services to patients and ability to train professionals and retain qualified personnel is critical for successful implementation efforts and in the delivery of high-quality services (17). Systems of rewards for new practices could also be a way to insure implementation in clinical settings. Severe workload, time pressure and pessimistic views of recovery for clients with psychosis were crucial barriers to implementation as well as (20). However, our questionnaire did not specifically examine these facilitating factors or obstacles encountered. It would be necessary to do this in the next studies on the implementation of the training. Training materials were recorded before the first wave of the COVID-19 pandemic, but the training test took place during the second wave between October and December 2020. The primary motivation for developing this online training was to respond to the many requests for training and seek an alternative to the lack of resources to provide these workshops. The evaluation took place in a period that accelerated the development of alternatives to the traditional training for clinical interventions that consisted of face-to-face workshops supplemented by manuals and clinical supervision (21). Movement restrictions linked to public health strategies to combat the pandemic probably had a positive effect on the high participation rate.

Data on the effects of e-learning in psychotherapeutic interventions are still scarce, and it is difficult to compare our results because the evaluation methods are very specific. The development of online training programs in the field of psychotherapeutic intervention education is growing (22) and uses either pre-experimental or quasi-experimental designs (23–25) or experimental design (26–29). These studies suggest that there is no difference in these learning outcomes when online and face-to-face teaching modalities; both modalities produce positive learning outcomes. According to group comparison studies, online training appears to be equal to or superior to textbook training in terms of knowledge acquisition (26, 27), which can be equal to face-to-face training (28) or superior (29). Regarding the application of course content in clinical simulations, in the study by Sholomskas et al. (26), online training was superior to textbook reading but inferior to face-to-face training, but online training was rudimentary and text-based in the following study. This superiority is repeated in a second study by Sholomskas and Carroll (27) who did not compare face-to-face training. In Dimeff et al.'s study (29) all three methods led to comparable improvements in clinicians' ability to apply course content in clinical simulations.

Compared to these data, the present study shows that knowledge about PEPS is improved, but we have no data on the effects of face-to-face training on knowledge acquisition. The SBI was improved in the study, but the data for face-to-face training showed a greater improvement than in the current study. It is possible that specifically practicing the ability to savor in a face-to-face group may have more impact than sitting alone in front of a computer. This should be investigated in future studies. This study did not measure the effects of training on the observation of clinical skills but only on feelings of competence. Future studies should use the observation of competence in a clinical simulation and fidelity check in real practice. The main limitations of this study are the lack of a control group and the lack of a behavioral measure to assess competence acquisition. A future study should offer post-training supervision sessions to increase the likelihood of implementation. The high representation of women in the sample could be identified as a limitation. However, in psychiatric care, psychologists, nurses, and occupational therapists are predominantly female. The sample reflects the clinical reality. Its main strength is that it makes PEPS facilitation training available at no cost at any time. Also, this study shows that this online training is accompanied by an improvement in the knowledge of program facilitation and the use of the skills taught in the everyday life of the participants.

The e-PEPS online training platform has aroused the interest of professionals in the field of psychiatry in French-speaking Switzerland, France, and Belgium. The training improves knowledge about PEPS and makes those who have taken it feel able to conduct the training in their place of practice. PEPS is predominantly conducted in places of living, day centers, and outpatient consultations. As a result of this study, training has been improved and is now freely available to all interested clinicians.

## Data Availability Statement

The raw data supporting the conclusions of this article will be made available by the authors, without undue reservation.

## Ethics Statement

Ethical review and approval was not required for the study on human participants in accordance with the local legislation and institutional requirements. The patients/participants provided their written informed consent to participate in this study.

## Author Contributions

AN, LF, and JF, in equal measure, conceptualized this research, recorded the training material, conceptualized e-PEPS, acquired, analyzed, interpreted the data, and drafted the first version of the manuscript. AK contributed to the technical management, the design of the platform, and critically revised the article for important intellectual content. All authors approved the final version for publication, agree to be accountable for all aspects of the work by ensuring that any questions related to its accuracy or integrity can be appropriately investigated, and resolved.

## Funding

This study was supported by a grant from the Fund for the Development of Prevention and Health Promotion of the Direction générale de la santé de l'Etat de Vaud.

## Conflict of Interest

The authors declare that the research was conducted in the absence of any commercial or financial relationships that could be construed as a potential conflict of interest.

## Publisher's Note

All claims expressed in this article are solely those of the authors and do not necessarily represent those of their affiliated organizations, or those of the publisher, the editors and the reviewers. Any product that may be evaluated in this article, or claim that may be made by its manufacturer, is not guaranteed or endorsed by the publisher.
